# Do age and mating status affect olfactory response of the parasitoid, *Microplitis croceipes* (Hymenoptera: Braconidae) to host-related plant odors?

**DOI:** 10.12688/f1000research.16927.2

**Published:** 2018-12-19

**Authors:** Matthew Burrows, Tolulope Morawo, Henry Fadamiro

**Affiliations:** 1 Entomology and Plant Pathology, Auburn University, Auburn, Alabama, 36849, USA; 2 926 Medical Detachment, US Army Medical Corps, Fort Benning, Georgia, 31905, USA

**Keywords:** cotton, electroantennogram, Heliothis virescens, physiological state, Y-tube olfactometer

## Abstract

**Background:** Parasitic wasps (parasitoids) use volatile organic compounds released by herbivore-infested plants to locate their hosts. Response of parasitoids to plant odors may be plastic and dependent on their physiological state. Using *Microplitis croceipes *(Hymenoptera: Braconidae), a relatively specialized larval endoparasitoid of *Heliothis virescens* (Lepidoptera: Noctuidae), we asked whether age and mating status of parasitoids affect their olfactory response to host-related odors.

**Methods:** Four odor stimuli of varying complexity were selected based on previous reports of parasitoid response to cotton volatiles: *cis*-3-hexenol (a green leaf volatile), α-pinene (a constitutive monoterpene), a 50/50 v/v binary mixture ( *cis*-3-hexenol + α-pinene), and *H. virescens*-infested cotton odors. Female *M. croceipes* used in Y-tube olfactometer bioassays were either mated or unmated, and grouped 1–3, 4–6, and 7–9 d-old. Female parasitoids used in electroantennogram (EAG) recording were mated and grouped 1–3, 4–6, 7–9 and 10–12 d-old.

**Results:** In Y-tube olfactometer bioassays, neither age nor mating status played a major role in the attraction of parasitoids to test odor stimuli, with two exceptions: 4–6 d-old mated parasitoids showed attraction to the binary mixture, and 1–3 d-old mated parasitoids showed attraction to *H. virescens*-infested cotton. Age did not affect EAG response of parasitoids to test stimuli.

**Conclusions:** The present results suggest that age and mating status do not play a major role in modulating olfactory responses of *M. croceipes* to host-related plant odors. Instead, plasticity of olfactory response may be limited in *M. croceipes* due to strong innate sensitivity to host-related odor cues.

## Introduction

Parasitic wasps (parasitoids) use volatile organic compounds (VOCs) released by herbivore-infested plants as odor cues to locate their hosts ^1– 4^. However, the level of olfactory response to VOCs may not remain consistent throughout the duration of parasitoid adult life. Changes in physiological states can affect response to VOCs in parasitoids and other insects ^5– 9^. Olfactory plasticity in insects has been previously attributed to changes in physiological states such as age, mating, and nutritional status ^6, 9– 13^.

Aging has been shown to affect olfaction in insects ^9, 14– 16^. This effect may be associated with senescence of olfactory structures or processing units in insects. For instance, behavioral senescence of responses involved with locomotion, olfaction, and learning has been reported in fruit flies ^17– 19^. However, previous studies have reported mixed results regarding age-related plasticity of response to VOCs in parasitoids, thus the need for further studies ^14, 20, 21^. Mating status of female parasitoids has also been reported to influence their foraging behavior and parasitization potential, with mated females showing a higher parasitization rate than unmated females ^13, 22, 23^. However, the effect of mating on plasticity of parasitoid response to host-related plant volatiles has gained little attention despite its implications and relevance to host searching and parasitization potential (but see Chen & Fadamiro ^24^).

In the present study, the relatively specialized larval endoparasitoid, *Microplitis croceipes* (Hymenoptera: Braconidae) and its caterpillar host, *Heliothis virescens* (Lepidoptera: Noctuidae) were used as a study system to test the effect of age and mating status on the olfactory response of parasitoids to host-related plant volatiles. *Heliothis virescens* is a generalist herbivore and a serious pest of cotton, tobacco, and other crops of economic importance ^25^. Based on previous reports on olfactory response in *M. croceipes* to cotton volatiles ^26– 30^, four odor stimuli of varying complexity were chosen. These include: *cis*-3-hexenol (a green leaf volatile), α-pinene (a constitutive monoterpene), a 50/50 v/v binary mixture ( *cis*-3-hexenol + α-pinene), and headspace volatiles from *H. virescens*-infested cotton. *cis*-3-Hexenol and α-pinene have been consistently detected in the headspace of *H. virescens*-infested cotton ^1, 29, 31^ and have been reported to elicit antennal and behavioral responses in *M. croceipes* ^24, 26, 29, 30^.

Y-tube olfactometer was used to test attraction (behavioral response) of parasitoids while electroantennogram (EAG) was used to record antennal response of female *M. croceipes* to select host-related cotton volatiles. To the best of our knowledge, this is one of the few studies that investigated the effects of age and mating status on olfactory responses of parasitoids to host-related plant volatiles. The implications of these findings are discussed.

## Methods

### Insects

Cocoons of *Microplitis croceipes* were provided by the USDA-ARS, Insect Biology and Population Management Research Laboratory (Tifton, Georgia, USA) and reared in our laboratory (Auburn University, AL, USA) on 2 ^nd ^– 3 ^rd^ instar larvae of *H. virescens*. Upon emergence, adult wasps were transferred to aerated plastic BugDorm® cages (Megaview Science Co. Taichung, Taiwan) and supplied with 10% sucrose/water solution (w/v). Naive (untrained) parasitoids were used in both EAG and Y-tube olfactometer bioassays to test innate responses of parasitoids to host-related plant odors. Eggs of *H. virescens* were initially purchased from Benzon Research Inc. (Carlislie, PA, USA) and reared in our laboratory at Auburn University. Larvae of *H. virescens* were reared on pinto bean artificial diet according to Shorey and Hale ^32^. In total, about 640 female parasitoids were used for bioassays. The general conditions for insect rearing and bioassays were 25 ± 1°C, 75 ± 5 % RH and L14:D10 h photoperiod.

### Plants

Cotton ( *Gossypium hirsutum*, var. max 9, All-Tex Seed Inc., Levelland, TX, USA) plants were grown in individual pots (9 cm high, 11 cm diameter) in a growth chamber at 26.6°C day, 25.6°C night, 60% RH, L16:D8 h (L:D) photoperiod. Seeds were planted in a top soil/vermiculate mixture. Plants used for headspace volatile collections were 4–6 weeks-old.

### Age and mating status treatments

Upon emergence, parasitoids were separated based on their sex. Subsequently, an equal number of females were randomly designated to ‘mated’ or ‘unmated’ cages. Male parasitoids were put inside cages (19 × 13 × 10 cm) designated to mated females at a 2:1 (male: female) ratio while cages designated to unmated females contained females only. Female parasitoids were allowed to mate for at least 24 h before use in bioassays. Both mated and unmated females were further designated to separate cages based on their age. Age groups 1–3, 4–6 and 7–9 d-old were used in Y-tube olfactometer bioassays while age groups 1–3, 4–6, 7–9, and 10–12 d-old were used in electroantennogram (EAG) recording. The 10-12 d-old age group was not included in behavioral bioassays due to low numbers of insects surviving beyond 10 days in the laboratory, resulting in a low number of replicates. It should be noted that Y-tube olfactometer bioassays required more replicates than EAG recording.

### Headspace volatile collection

Headspace volatiles were collected from *H. virescens*-infested cotton plants using the protocol described by Ngumbi *et al*. ^31^. A plant pot with soil was wrapped with aluminum foil to minimize contamination. The plant was then placed in a volatile collection chamber (Analytical Research Systems, Inc., Gainesville, FL, USA) consisting of a 5-L air-tight glass jar. A purified air stream of 500 ml/min was passed through the jar at room temperature using Teflon tubing connected to an air delivery system. To induce volatiles from plants, 30 2 ^nd ^–3 ^rd^ instar larvae of *H. virescens* were allowed to feed on a cotton plant for 24 h during volatile collection from 1100 h one day to 1100 h of the following day. Headspace volatiles were collected with a trap containing 50 mg of Super-Q (CAT#: 2735, Alltech Associates, Deerfield, IL, USA) and eluted with 300 µl of methylene chloride. The resulting extract was stored in a freezer (-20°C) until use.

### Y-tube olfactometer bioassays

A Y-tube olfactometer (Analytical Research Systems, Inc., Gainesville, FL, USA) was used to test attraction of female *M. croceipes* to four odor stimuli of varying complexity. The setup and procedure was similar to that reported by Morawo and Fadamiro ^33^. Parasitoids were introduced individually into the olfactometer and allowed to make a choice between test stimulus and control. Insects were tested once and discarded. Parasitoids that made no choice within 5 min were removed and excluded from the analyses. The number of non-responding parasitoids in the 24 sets of bioassays ranged from 0 to 5 with a mean of 1.2 insects per test.

*cis*-3-Hexenol (CAT#: W256307) and α-pinene (CAT#: 147524) (purity 95-99%; Sigma-Aldrich®, St. Louis, MO, USA) were individually formulated in hexane (HPLC-grade) at 1 µg/µl concentration. A 50/50 v/v binary mixture ( *cis*-3-Hexenol + α-pinene) of the two compounds was also prepared. A central dose of 10 µg (10 µl sample) was previously determined to be optimal in a related study ^30^. In separate bioassays, each compound or binary mixture was delivered as a 10 µl sample on filter paper strips (40 × 7 mm, CAT#: 1001090, Whatman® No. 1) in the treatment arm while the control arm contained the same volume of hexane (solvent control). Humidified and purified (charcoal filtered) air was pushed into each arm at the rate of 250 ml/min and removed by suction from the central arm of the olfactometer at the rate of 500 ml/min to avoid odor mix-up.

To test parasitoid response to *H. virescens*-infested cotton, one arm of the olfactometer was connected to an air-tight glass jar (5-L) containing an infested plant (with host larvae). The other arm was connected to a similar glass jar containing a pot of soil covered with aluminum foil, which served as control. A new plant was used on different days during which bioassays were conducted (5–7 plant replicates). Inlet air was pushed into the olfactometer through each jar at the rate of 300 ml/min and sucked out at the rate of 600 ml/min. Experiments were performed in a randomized complete block design with equal number of insect replicates from each age and mating status groups tested per day ( *n* = 20 per test). All olfactometer bioassays were conducted between 1100 h and 1700 h on different days.

### Electroantennogram recording (EAG)

EAG response of female *M. croceipes* was recorded to measure odor perception in mated parasitoids. The EAG protocol used was previously described by Ngumbi *et al*. ^31^. Glass capillaries (1.1 mm I.D.) filled with Ringer solution served as reference and recording electrodes. The reference electrode was connected to the back of the head of a female *M. croceipes* while the recording electrode was connected to the cut tip of the terminal segment of the antenna. The analog signal was detected through a probe (INR-II, Syntech, the Netherlands), and was captured and processed with a data acquisition controller (IDAC-4, Syntech, the Netherlands). EAG 2000 software v2.7 (Syntech, the Netherlands) was used to analyze digital signal readouts. Test stimuli were delivered as 10-µl samples (10 µg dose) on filter paper strips (7 × 40 mm) placed inside 14 cm Pasteur pipettes (Fisher Scientific, Pittsburgh, PA, USA).

Four treatment stimuli and two control stimuli were individually delivered as 0.2-s puffs of air, with 2 min interval between puffs. For each antennal preparation, the following stimuli were presented: hexane (control), methylene chloride (control), *cis*-3-hexenol, α-pinene, binary mixture, headspace volatile extract, hexane and methylene chloride. Thus, hexane and methylene chloride (solvent controls) were applied at the beginning and end of each recording series while the position of other test stimuli was randomized across replicates (see Morawo *et al.* ^34^). Recordings were performed in a randomized complete block design with equal number of insect replicates ( *n* = 10) from each age group tested per day.

### Data analyses

Attraction of parasitoids to each of four test stimuli in Y-tube olfactometer was modeled as a binary response (stimulus = 1, control = 0) using logistic regression to analyze possible interactions between age and mating status factors. The model adequacy for each set of experiment was confirmed with a likelihood ratio test ^35^. When no significant interaction was recorded, each factor was analyzed separately. For olfactometer data, deviation of parasitoid responses from a 50:50 (stimulus: control) distribution was analyzed using a Chi-square goodness-of-fit test. Absolute EAG responses (EAG response to solvent control deducted from EAG response to test stimuli) of mated parasitoids across age groups were compared using Kruskal-Wallis test. All analyses were performed in SAS v9.2 (SAS Institute Inc., Cary, NC, USA) with *P* = 0.05 level of significance.

## Results

### Effect of age and mating on attraction of parasitoids in Y-tube olfactometer

**Figure 1.**
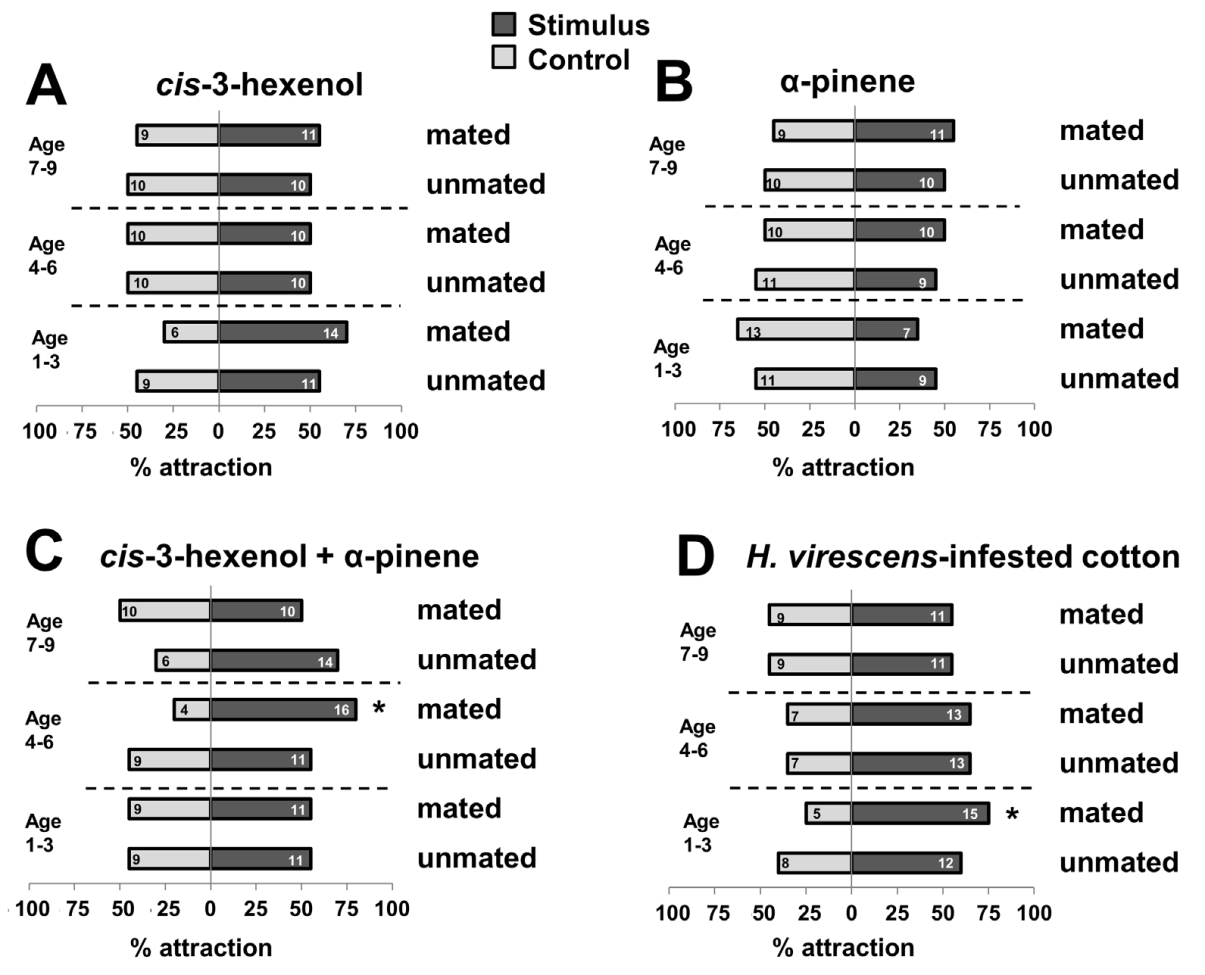
Effect of age and mating status on the attraction of female *Microplitis croceipes* to four host-related plant odors in Y-tube olfactometer bioassays. Bars represent percentage of mated and unmated parasitoids of ages 1–3, 4–6, and 7–9 d-old when given a choice between hexane (solvent control) and synthetic compounds *cis*-3-hexenol ( **A**), α-pinene ( **B**), a 50/50 v/v binary mixture of *cis*-3-hexenol and α-pinene ( **C**), and a choice between control jar (with no plant) and *Heliothis virescens*-infested cotton ( **D**). Synthetic compounds were formulated in hexane at 1 µg/µl and presented as 10 µl samples (10 µg dose). Thirty 2 ^nd^–3 ^rd^ instar larvae of *H. virescens* were allowed to infest cotton plants for 24 h before bioassays. *N* = 20 responding parasitoids per choice test. Numbers in the bars indicate actual number of responding individuals that chose each arm of the olfactometer. Asterisk (*) indicates significant deviation of parasitoid responses from a 50:50 (stimulus: control) distribution ( *χ* ^2^ goodness of fit test, *P* < 0.05).

Overall, there was no significant interaction between age and mating status factors for any of the four odor stimuli tested ( *cis*-3-hexenol: *P* = 0.7295, Figure 1A; α-pinene: *P* = 0.7352, Figure 1B; binary mixture: *P* = 0.1136, Figure 1C; host-infested cotton: *P* = 0.7044, Figure 1D; Logistic Regression). However, mated parasitoids, 4-6 d-old were significantly (80/20%, *χ* ^2^= 7.20, *df* = 1, *P* =0.0073) more attracted to the binary mixture ( *cis*-3-hexenol + α-pinene) compared to hexane control ( Figure 1C). Similarly, 1-3 d-old mated parasitoids were significantly (75/25%, *χ* ^2^= 5.00, *df* = 1, *P* =0.0253) more attracted to *H. virescens*-infested cotton compared to control ( Figure 1D). Parasitoids did not show significant attraction to test stimuli in other Y-tube olfactometer bioassays.

### Effect of age on EAG response of parasitoids

**Figure 2.**
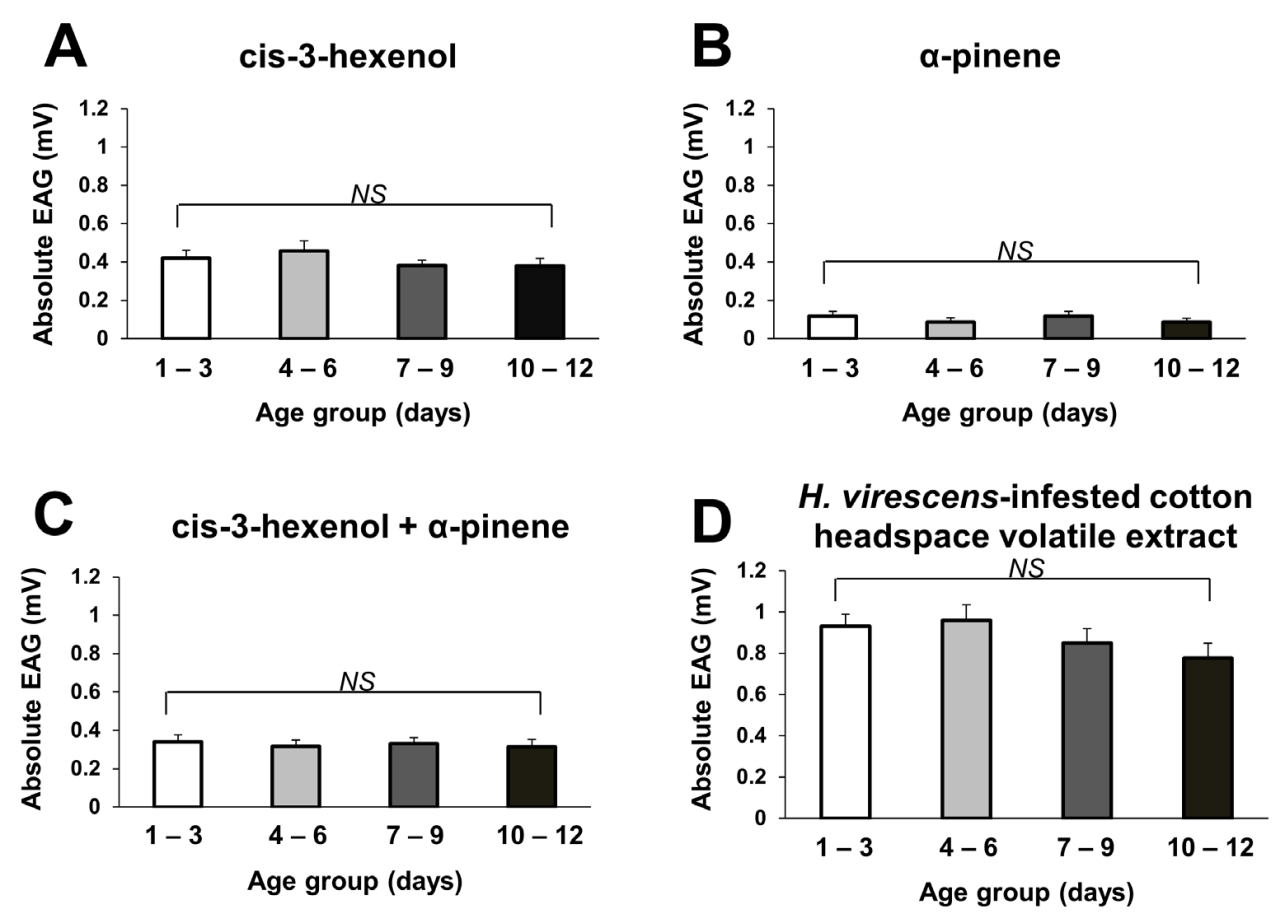
Effect of age on the electroantennogram (EAG) response of mated female *Microplitis croceipes* to four host-related plant odors. Bars represent mean absolute EAG responses (mV± SE, *N* = 10) of mated parasitoids age 1–3, 4–6, 7–9 and 10–12 d-old to *cis*-3-hexenol ( **A**), α-pinene ( **B**), a 50/50 v/v binary mixture of *cis*-3-hexenol and α-pinene ( **C**), and *Heliothis virescens*-infested cotton headspace volatile extract ( **D**). Absolute EAG for each stimulus is the actual EAG value minus EAG value of solvent control. Synthetic compounds were formulated in hexane at 1 µg/µl and presented as 10 µl samples (10 µg dose). *NS* indicates no significant difference.

In general, the age of mated female *M. croceipes* did not have a significant effect on their EAG response to test odor stimuli ( Figure 2). Both relatively young and older parasitoids showed similar levels of innate antennal sensitivity to single components, binary mixture and headspace volatile extract of host-infested cotton. These results are mostly in agreement with those recorded in Y-tube olfactometer bioassays.

Attraction of female Microplitis croceipes to four host-related plant odors in Y-tube olfactometer bioassays Click here for additional data file. Copyright: © 2018 Burrows M et al. 2018 Data associated with the article are available under the terms of the Creative Commons Zero "No rights reserved" data waiver (CC0 1.0 Public domain dedication).

Electroantennogram (EAG) response of female Microplitis croceipes to four host-related plant odors Click here for additional data file. Copyright: © 2018 Burrows M et al. 2018 Data associated with the article are available under the terms of the Creative Commons Zero "No rights reserved" data waiver (CC0 1.0 Public domain dedication).

## Discussion

Age and mating status are among several physiological factors that may affect olfactory responses of parasitoids to odor cues used in foraging. In the present study, neither age nor mating status of female *M. croceipes* played a major role in their olfactory responses to host-related plant volatiles. In general, relatively younger parasitoids showed similar levels of attraction to test stimuli as older parasitoids. Likewise, mated and unmated female parasitoids showed little or no difference in their attraction to host-related odors in Y-tube olfactometer bioassays. Subsequent experiments were conducted to measure the level of odorant perception in mated female parasitoids across age groups. The results showed that EAG response of mated parasitoids was not significantly different across age groups. 

Although aging may negatively affect the host searching ability of female parasitoids, it may not always be attributed to a decline in odor perception in all species. Previous studies showed that relatively younger parasitoids tend to parasitize at a significantly higher rate than older parasitoids ^36– 39^. This may be in part due to senescence of some odorant processing apparatus or due to a decrease in energy levels in older parasitoids, thus affecting foraging activities. In a previous related study, age did not affect EAG response of *M. croceipes* to single VOCs ^21^. In instances where age played a significant role, plasticity of olfactory response in insects at the peripheral and behavioral levels has been attributed to senescence through physiological and neuronal mechanisms ^17, 19^.

A few previous studies have reported that the mating status of female parasitoids may affect their foraging behavior and parasitization potential ^13, 22, 23, 40^. In the present study, mating status of female *M. croceipes* had no significant effect on their olfactory response to host-related odors. This suggests that both virgin and mated females of *M. croceipes* are likely to seek hosts using host-related odor cues. The concepts of haplodiploidy and optimal foraging provide a better understanding of the ecological ramifications of these results. Haplodiploid parasitoids such as *M. croceipes* produce male-only offspring from unfertilized eggs and male/female offspring from fertilized eggs. If the sex ratio of a local population is already at equilibrium, host foraging by unmated females may yield immediate benefits ^41^. Otherwise, the cost may outweigh immediate benefits with the development of a male-biased population. Mated females are expected to optimize host foraging and produce progeny with a more balanced sex ratio, which is a critical fitness benefit for the population.

Overall, age and mating did not significantly affect the attraction of female *M. croceipes* to four test stimuli of varying complexity in the present study, with a few exceptions: mated parasitoids, 4–6 d-old showed significant attraction to the binary mixture and 1–3 d-old mated parasitoids showed significant attraction to *H. virescens*-infested cotton. The degree of host specificity in *M. croceipes* may provide a plausible explanation for the overall pattern and exceptions recorded in the present study. It has been proposed that specialist parasitoids exhibit strong congenitally fixed responses while generalist parasitoids exhibit greater plasticity of response to host-related cues ^42^. This hypothesis is consistent with the present results in which *M. croceipes* (specialist) showed little olfactory plasticity with changes in physiological state. However, *M. croceipes* is not a strictly specialized species at the extreme of the spectrum. Instead, *M*. *croceipes* is a relatively specialized parasitoid utilizing *Heliothis*/ *Helicoverpa* host species ^43^. This may possibly explain the few exceptions in which mated and relatively young parasitoids showed significant attraction to the binary mixture and host-infested cotton.

In summary, the current findings suggest that age and mating status do not play a major role in modulating olfactory responses of *M. croceipes* to host-related odors. Instead, plasticity of olfactory response may be limited in *M. croceipes* due to a strong innate sensitivity to host-related odor cues. This may have an impact on their potential as biological control agents. Other physiological factors such as level of nutrition may also have significant effect on olfactory plasticity in parasitoids ^11, 44^. This creates an opportunity for augmentation of parasitoids after field releases. Future studies, especially in the field, should investigate the effect of other physiological conditions that may affect plasticity of behavioral response to host-related odors in natural enemies.

## Data availability

The data referenced by this article are under copyright with the following copyright statement: Copyright: © 2018 Burrows M et al.

Data associated with the article are available under the terms of the Creative Commons Zero "No rights reserved" data waiver (CC0 1.0 Public domain dedication). 

The work presented here was part of an MS project completed by MB. The results presented here have been previously published as MB’s MS thesis available from Auburn University Electronic Theses and Dissertations repository: http://hdl.handle.net/10415/5108

F1000Research: Dataset 1. Attraction of female *Microplitis croceipes* to four host-related plant odors in Y-tube olfactometer bioassays, https://doi.org/10.5256/f1000research.16927.d224669 ^45^

F1000Research: Dataset 2. Electroantennogram (EAG) response of female *Microplitis croceipes* to four host-related plant odors, https://doi.org/10.5256/f1000research.16927.d224670 ^46^
