# Supplementary material for: Do age and mating status affect olfactory response of the parasitoid, Microplitis croceipes (Hymenoptera: Braconidae) to host-related plant odors?
Source: F1000Res. 2018 Dec 19;7:1817. Originally published 2018 Nov 20. [Version 2] doi: 10.12688/f1000research.16927.2 (PMC6283379; doi:10.12688/f1000research.16927.2)
Supplement: Peer review history

# Peer review: Referee response for version 1

**DOI:** 10.5256/f1000research.18508.r41225

**Published:** 2018-12-05

**Authors:** Andrea Clavijo McCormick

**Affiliations:** 1 Institute of Agriculture and Environment, Massey University, Palmerston North, New Zealand

The article by Burrows and coworkers investigates the olfactory responses of mated and unmated *Microplitis croceipes* females of different age-groups to host-related plant volatiles. The results indicate that age and mating status do not affect the odour-guided behaviour or EAG responses of the parasitoids.

 The paper is clear and well written, citing relevant literature on the topic from previous studies by the same group and other authors. The methods used are standard in the field of chemical ecology and appropriate to answer the research question. One minor issue regarding the methods section is the inclusion of the conditions in which behavioural trials were conducted. 

 Temperature and relative humidity are known to affect insect behaviour; therefore it would be important to mention if the assays were conducted under controlled conditions or not. And if the second was true, which were the average values for these parameters on the assayed days.

 A couple of minor style comments:

1. The use of 'and/or' in the introduction (second paragraph). It is a matter of preference but 'or' only should be sufficient.
2. In the methods section (Insects) 'L14:D10 h (L:D) photoperiod' is repetitive, either use L14:D10 h photoperiod or 14:10h L:D photoperiod.

The statistical analyses are correct and appropriate to the data collected. The data sets provided ensure the reproducibility of the study, and the results support the conclusions.

 This is a high quality manuscript and I recommend it for indexing without hesitation.

I have read this submission. I believe that I have an appropriate level of expertise to confirm that it is of an acceptable scientific standard.

# Response

**Published:** 2018-12-17

**Authors:** Tolulope Morawo

**Affiliations:** Auburn University, USA

We thank the reviewer for the comments on the manuscript. We have indicated that the same general conditions (25 ± 1°C, 75 ± 5 % RH) used for insect rearing were maintained during bioassays. The other two minor changes have been made as suggested.

# Peer review: Referee response for version 1

**DOI:** 10.5256/f1000research.18508.r40848

**Published:** 2018-11-30

**Authors:** Lukasz Stelinski

**Affiliations:** 1 Department of Entomology and Nematology, Citrus Research and Education Center, University of Florida, Lake Alfred, FL, USA

This manuscript describes a set of behavioural and electrophysiological experiments testing the hypotheses that: 1) mating status and 2) age affect behavioural and antennal response of the parasitoid wasp, *Microplitis croceipes* to host-related plant odors. Both synthetic and authentic host related odors were tested. Odors were either a general green leaf volatile or volatile organic compounds induced by host ( *Heliothis virescens*) feeding on plants. Overall, the data tend to reject both hypotheses. The authors postulate that *M. croceipes *display strong innate sensitivity and response to host-related odors with little plasticity due to their relative ecological host specialization.

 The investigators have made numerous scientific contributions using this parasitoid-host study system over a number of years and thus their methods are well established and proven state of the art. The experiments were adequately replicated and the methods are robust and well designed to test the stated hypotheses. The data analysis is appropriate. The data is clearly presented and the conclusions are consistent with the results obtained. I have no reservations endorsing this manuscript for indexing.

I have read this submission. I believe that I have an appropriate level of expertise to confirm that it is of an acceptable scientific standard.

# Response

**Published:** 2018-12-17

**Authors:** Tolulope Morawo

**Affiliations:** Auburn University, USA

We thank the reviewer for the comments on the manuscript.
